# High silent prevalence of human herpesvirus 1 (HSV-1) infection affecting the indigenous reservation of the municipality of Dourados, Central-West Brazil

**DOI:** 10.1186/s12879-024-09497-5

**Published:** 2024-07-17

**Authors:** Flávia Freitas de Oliveira Bonfim, Livia Melo Villar, Julio Croda, Jéssica Gonçalves Pereira, Ana Carolina Silva Guimarães, Solange Rodrigues da Silva, Crhistinne Cavalheiro Maymone Gonçalves, Lucas Fernando Tinoco Leonardo, Grazielli Rocha de Rezende Romeira, Gabriela Alves Cesar, Sabrina Weis-Torres, Vivianne de Oliveira Landgraf de Castro, Marco Aurélio Horta, Simone Simionatto, Ana Rita Coimbra Motta-Castro, Vanessa Salete de Paula

**Affiliations:** 1 Molecular Virology and Parasitology Laboratory, Oswaldo Cruz Foundation, Rio de Janeiro, 21040360 Brazil; 2 Viral Hepatitis Laboratory, Oswaldo Cruz Foundation, Rio de Janeiro, 21040360 Brazil; 3 Department of Epidemiology of Microbial Diseases, Universidade Federal de Mato Grosso do Sul-UFMS, Campo Grande, Mato Grosso do Sul Brazil; 4 Fiocruz Mato Grosso do Sul, Fundação Oswaldo Cruz, Campo Grande, Mato Grosso do Sul Brazil; 5 Department of Epidemiology of Microbial Diseases, Yale School of Public Health, New Haven, CT USA; 6 Binacional Campus of Oiapoque, Federal University of Amapá, Amapá, 68903419 Brazil; 7 Center for Biological and Health Sciences, Blood Center Sector, Federal University of Mato Grosso do Sul, Campo Grande, Grosso do Sul 79070900 Brazil; 8 State Department of Health of Mato Grosso do Sul, Campo Grande, Mato Grosso do Sul 79031350 Brazil; 9 Laboratory of Tryposomatid Biology, Oswaldo Cruz Foundation, Rio de Janeiro, 21040360 Brazil; 10 Oswaldo Cruz Foundation, Biosafety Level 3 Facility (BSL-3), Rio de Janeiro, 21040360 Brazil; 11 Health Sciences Research Laboratory, Federal University of Grande Dourados (UFGD), Dourados, Mato Grosso do Sul Brazil; 12 Environmental Analysis Laboratory, Southern Cross University, Military Road East, Lismore, NSW 2480 Australia

**Keywords:** *Human alphaherpesvirus* 1, Human herpes virus 1, Indigenous, Brazil, Epidemiology

## Abstract

**Background:**

The indigenous population located in the central region of Brazil, is the second largest in terms of population size in the country. The Indigenous Reserve of Dourados has risk factors that increase the vulnerability of the indigenous population to infectious diseases, especially *Human alphaherpesvirus* (HSV-1), a neglected disease with high prevalence in priority populations in developing countries. The virus can also cause many more severe diseases, including widespread neonatal infections, herpetic keratitis, and herpes encephalitis, which can be fatal if left untreated. We estimated the prevalence of anti-HSV-1 antibodies and correlated it with the demographic and behavioral characteristics of the Indigenous population of the Jaguapirú and Bororó villages (Dourados, Mato Grosso do Sul (MS), Brazil).

**Methods:**

Our approach was cross-sectional. From March 2017 to November 2018. Using anti-HSV-1 (Gg1) IgM and anti-HSV-1 (gG1) IgG Euroimmun and the detection and quantification of HSV-1 viral load in plasma samples, through real-time PCR. The maps were constructed using QGIS and the statistical analyses using R Studio software.

**Results:**

A total of 1138 individuals (> 18 years old) were enrolled. The prevalence of anti-HSV-1 IgM and IgG were 20% and 97.5%, respectively. The prevalence of anti-HSV-1 antibodies for IgG was higher in both sexes. Anti-HSV-1 IgM antibodies were present in 17.1%, 21.2%, 12.5%, and 22% of the participants with urinary problems, genital wounds, genital warts, and urethral discharge, respectively. Real-time PCR was used for confirmatory testing; HSV-1 DNA was detected in 25.6% (54/211) of anti-HSV1 IgM-positive samples. Viral loads ranged from 5.99E + 02 to 3.36E + 13.

**Conclusions:**

The seroprevalence of HSV-1 IgM and detection of HSV-1 DNA in the Indigenous population confirmed high silent prevalence. Furthermore, the seroprevalence of HSV-1 in the Indigenous population was higher than that reported in the general adult Brazilian population. Various socioeconomic factors, drug use, and health and sexual behaviors could contribute to the facilitation of HSV-1 transmission in the Indigenous population. Our results may help develop culturally appropriate intervention programs that eliminate health access barriers and improve the implementation of public health policies aimed at promoting information regarding the prevention, treatment, and control of HSV-1 infection in Brazilian Indigenous populations.

## Introduction

The Indigenous “priority population” in the state of Mato Grosso do Sul (MS) is at increased risk of acquiring *Human Alphaherpesvirus* 1 (*Human herpes virus* 1 or HSV-1). According to the United Nations Declaration on the Rights of Indigenous Peoples, adopted through resolution A/RES/61/295 by the United Nations General Assembly, firmly affirms the equal entitlement of Indigenous communities to the utmost possible standard of physical and mental well-being. This includes their right to access quality healthcare services to safeguard their health needs and uphold their dignity [1]. The lack of information regarding HSV-1 prevalence is a priority when it comes to unique health behavior, awareness, and prevention. According to the 2010 census (conducted by the Brazilian Institute of Geography and Statistics (IBGE) and the National Indian Foundation (FUNAI), Brazil has an Indigenous population of approximately 896,917 individuals, with 324,834 living in urban areas and 572,083 living in rural areas; Currently about 1.3 million Indigenous people in Brazil. Indigenous people in Brazil include 305 ethnically diverse groups and 274 languages, spread over 12.5% of the Brazilian geographic area. The Indigenous population studied in Mato Grosso do Sul, located in the central region of Brazil [2] is the second largest in Brazil [2, 3].

Inhabiting the Jaguapirú and Bororó villages of the city of Dourados in Mato Grosso do Sul (MS), the largest ethnic groups are Guarani-Kaiowá and Terena, comprising approximately 18,000 people in an area of 3474.50 hectares [4, 5]. In addition, the Atikum, Kini Kinawa, Kadiwéu, Guató, Guarani-Nhandeva, and Ofaié ethnic groups inhabit the entire territory of MS [3–5]. The geolocation of Indigenous populations places them in close proximity to urban centers and international land borders. In turn, this increases interactions with non-Indigenous populations, and predisposes them to alcoholism and drug use. Additionally, Indigenous populations have low socioeconomic and educational levels related to cultural rituals, and risky sexual behaviors such as lack of condom use, unprotected sex, and a history of sexually transmitted infections (STIs), worsened by absent or limited access to health care and information. Further, several cultural rituals require the use of shared and inadequately disinfected sharp objects. These risk factors increase the vulnerability of the Indigenous population of the Dourados/MS area to infectious diseases, specifically HSV-1 [6–11]. The scarcity of specific epidemiological data on STIs from indigenous Brazilian communities greatly contributes to the higher morbidity and mortality rates of HSV-1 in these areas compared to the general population [11, 12].

The Herpesviridae family of enveloped DNA viruses including nine human herpesviruses (HSV) widely distributed worldwide. HSV-1 is a double-stranded DNA virus, transmitted through mucosal epithelial cells by breaks in the skin. It migrates to nervous tissue, where it persists in a latent state. In most cases it is localized to orofacial lesions in the trigeminal ganglia, and can reactivate at any time [13]. Most infections begin during childhood or youth via direct oral contact with secretions contaminated with viral particles. Lytic infection, in most cases, can cause mild illnesses such as cold sores and oral lesions (herpes labialis) [14, 15]. However, HSV-1 has been found to concomitantly infect the orofacial areas and the genital tract [13]. The virus is also capable of causing many more serious diseases, such as herpetic keratitis [16], herpes encephalitis, which can be fatal if left untreated [17, 18], and disseminated neonatal infections [19]. HSV-1 is being studied as a major etiological factor Alzheimer’s disease [18–20]. HSV-1 is also closely related to HSV-2, a human pathogen with an increased risk with human immunodeficiency virus-1 (HIV-1) infection [21, 22]. Reactivation can occur in response to various stimuli, such as immunosuppression, stress, or hormonal changes, which are symptomatic; however, transmission can also occur in an asymptomatic form [23–26]. Latent reactivation leads to asymptomatic or generalized episodes [26–30].

HSV-1 is a highly prevalent human pathogen. About 3,583.5 million of the global population aged 0–49 years have been infected orally with HSV-1 and its worldwide prevalence is approximately 63.6%. Genital HSV1 is prevalent in 5.2% of individuals aged 15–49 years worldwide. Simultaneous oral and genital infections are present in 66.6% of the population, of which 3,752.0 million people aged 0–49 years were infected globally. Moreover, 3.3% of individuals aged 15–49 years had genital HSV-1 and reported oral sex in the year prior (2016) [31, 32]. A study conducted in Latin America and the Caribbean revealed that the mean seroprevalence of HSV-1 was 57.2% among healthy adult populations and 90.9% among adult populations with comorbidities; in patients with clinically diagnosed genital ulcers, the average seroprevalence was 0.9%, and genital herpes, the average seroprevalence was 10.9% for HSV-1, due to other etiologies that generate genital ulcers [33]. The seroprevalence of HSV-1 antibodies in the general population of Brazil is 67.2%, accounting for a total of 1,090 individuals aged 1–40 years, and the overall seroprevalence among men and women is 66.2% and 68.2%, respectively and among Brazilian cities, the seroprevalence of HSV-1 was 73.3% in Manaus, 68.6% in Rio de Janeiro, 62.1% in Porto Alegre, and 47.0% in Fortaleza [34].

We aimed to estimate the prevalence and analyze factors associated with HSV-1 infection in the Indigenous population of the Dourados/MS Reserve using HSV-1 antibody from participants in the Jaguapirú and Bororó villages. Our research is useful for developing culturally appropriate programs that can facilitate access to public health services, eliminate stigmas regarding HSV-1 transmission and treatment, and support the implementation of public health policies to promote the prevention, treatment, intervention, and control of HSV-1 infections in the Brazilian Indigenous population.

## Materials and methods

### Ethical statement

All participants provided informed consent before participating in the study. The study adhered to the principles of the Declaration of Helsinki. Approval was received from the Ethics Committee of the University of Grande Dourados (UFGD-MS) in March 2017 (CA AE:62012616.3.0000.5160 (number 2.000.496)). This study was conducted with utmost professionalism and care, ensuring that all doubts and concerns were addressed promptly.

### Sampling

The Dourados Indigenous Area Health Team (EMSI, Bororó, and Jaguapiró, 1 and 2) provided multidisciplinary expertise and support for this study. Our sampling team of doctors and nurses q1the use of sterile equipment and needles for venous blood draws.

Our approach was cross-sectional. From March 2017 to November 2018, a team of skilled health professionals consisting of medical doctors, physicians, biologists, and nurses, with the assistance of a proficient local interpreter, collected data and blood samples from the Indigenous population from two villages in Dourados, Mato Grosso do Sul (MS), Brazil. The research population was made up exclusively of indigenous adults aged 18 or over who were able to provide informed consent. Before participation, each individual voluntarily signed a comprehensive consent form and responded to a personalized socio-epidemiological survey, ensuring an optimal level of privacy and anonymity. To further validate our structured questionnaire, we sought input from Indigenous health professionals and an Indigenous health agent translated the questionnaire into the native language when needed.

The questionnaire-based interview aimed to capture a comprehensive profile of the participants, focusing on their risks and protective factors. We carefully selected information on risk behaviors for HIV and HBV, including sociodemographic information such as income, education status, and housing conditions, substance use history, medical history, and signs and symptoms of hepatitis B and C, HIV, and HSV infections. The study included consenting individuals aged 18 years who resided in the study area. Participants who failed to provide sufficient blood samples for anti-HSV-1 testing were excluded.

The study population was determined using data from the Special Indigenous Health District of Mato Grosso do Sul (SIHD/MS). According to SIHD/MS, there are 13,094 Indigenous individuals residing in Bororó and Jaguapirú within the Dourados/MS municipality, with 6291 over the age of 18. Factoring in a 20% loss due to refusal, the estimated eligible population for sampling was 3400, comprising both males and females, and individuals from the Guaraní-Kaiowá, Terena, Kadiwéu, and Guarani-Nhandeva ethnic groups. The selection criterion for the regression analysis to calculate the p-value, the POR (95% CI) above 0.20 was statistically calculated. The eligible sample size for our study included 295 individuals, comprising both men and women. We recruited 1,168 study participants over the age of 18 who agreed to take part in the survey; the remainder did not agree to take part in the survey, around four times more than the estimated sample size required. This represents 8.9% of the total population living in the Dourados Indigenous Reserve/MS.

### Serological analysis

Anti-HSV-1 (gG1) IgM and anti-HSV-1 (gG1) IgG Euroimmun (Euroimmun, a PerkinElmer Company, Medizinische labordiagnostika AG-Germany: Anti-HSV-1 (gG1) ELISA (IgM) order no. EI 2531-9601-2 M LOTE: E180321AT and Anti-HSV-1 (gG1) ELISA (IgG) order no. El 2531-9601-2 G LOTE: E220906BL) ELISA (enzyme-linked immunosorbent assays) detected serological markers of HSV-1 infection. The sensitivity and specificity of both immunoassays were determined according to the manufacturer’s instructions using positive, calibrator, and negative controls. The test had a specificity of 100% and a sensitivity of 100%. The results were assessed using binary outcomes of positive and negative HSV-1 IgG and IgM. The ELISA technique ensured high antigen specificity, which minimized the incidence of false positives due to cross-reactions with anti-HSV-2. Therefore, it can be concluded that this assay is reliable and accurate as per the protocol.

### Detection and quantification of HSV-1 viral load by qPCR UL 39

Detection and quantification of the viral load of HSV-1 in the plasma samples were performed using real-time PCR (qPCR). The TaqMan oligonucleotides and probes used in this study are listed. Single qPCR was performed in a reaction mix comprising 1 µL of 25x PCR enzyme (Life Technologies mix, California, USA), 1.25 µL of each oligonucleotide (1 µM), 1 µL probe (0.4 µM), 6 µL of 2x PCR Buffer (Life Technologies, California, USA) and 3 µL of DNA. Absolute quantification was performed using a previously described synthetic standard curve [35]. In order to quantify the viral load, DNA samples were evaluated by qPCR using AgPath-ID™ One-Step RT-PCR Kit (Thermo Fisher, Scientific), according to manufacturer’s instructions to allow detection and quantification of HSV-1 UL39. Cycling conditions were as follows: initial activation 50°C for 2 min, denaturation and Enzyme Activation at 95°C for 10 min, followed by 40 cycles of denaturation at 95°C for 15 s, hybridization and extension at 60°C for 1 min. The primers for qRT-PCR were the primers for qRT-PCR were Primers (Sense): 5´- CCAACTGCACCATGATCATCGA − 3´, Primers (Anti-sense): 5´- GATGTTTGTCACCGCAACGAA − 3´ and Probe FAM 5’-CCCGCGCACCACGTC-3’ MGB [35–37].

### Statistical analysis

Information was categorized into distinct categories, referred to as blocks. Block A encompassed general data, Block B covered sociodemographic information, Block C delved into the history of drug and alcohol usage, Block D focused on tuberculosis, Block E examined STI status, and Block F recorded the tests performed. The participants responded to the questionnaires in a “yes” or “no” format. This questionnaire was validated by the Ethics Committee of the UFGD-MS and is included in Supplementary Information. Demographic data, including age, sex, and socioepidemiological information, were analyzed using Pearson’s chi-square test to evaluate their correlation with serological status. Prevalence was established, and the prevalence odds ratio (POR) were estimated to determine the association between sociodemographic variables and HSV-1 positivity among Indigenous people. We assessed the relationship between age, sex, socioeconomic determinants, and HSV-1 infection using Pearson’s chi-square test. Logistic regression was used to estimate the Prevalence odds Ratio (POR). The significance level was set at *p* < 0.05. Analyses were performed using RStudio software (version 2023.03.0). Maps were constructed using QGIS (version 3.26.1).

## Results

In this study, we analyzed 29.1% (1168) of the eligible population over the age of 18 years. Females consisted of a larger proportion of the sample at 80.9% (945/1168), while the males comprised 19.1% (223/1168). The age of the participants ranged from 18 to 103 years for women and 18 to 83 years for men. A high overall prevalence of HSV-1 IgG was observed (97.5%), and in females and males (97.6% and 97.3%, respectively), as shown in Table 1. The distribution of HSV-1 positivity and negativity corresponding to age, where the mean age at infection was 30 years (interquartile range 22.40) years, with the highest HSV-1 prevalence between 29 and 39 years.

**Fig. 1:**
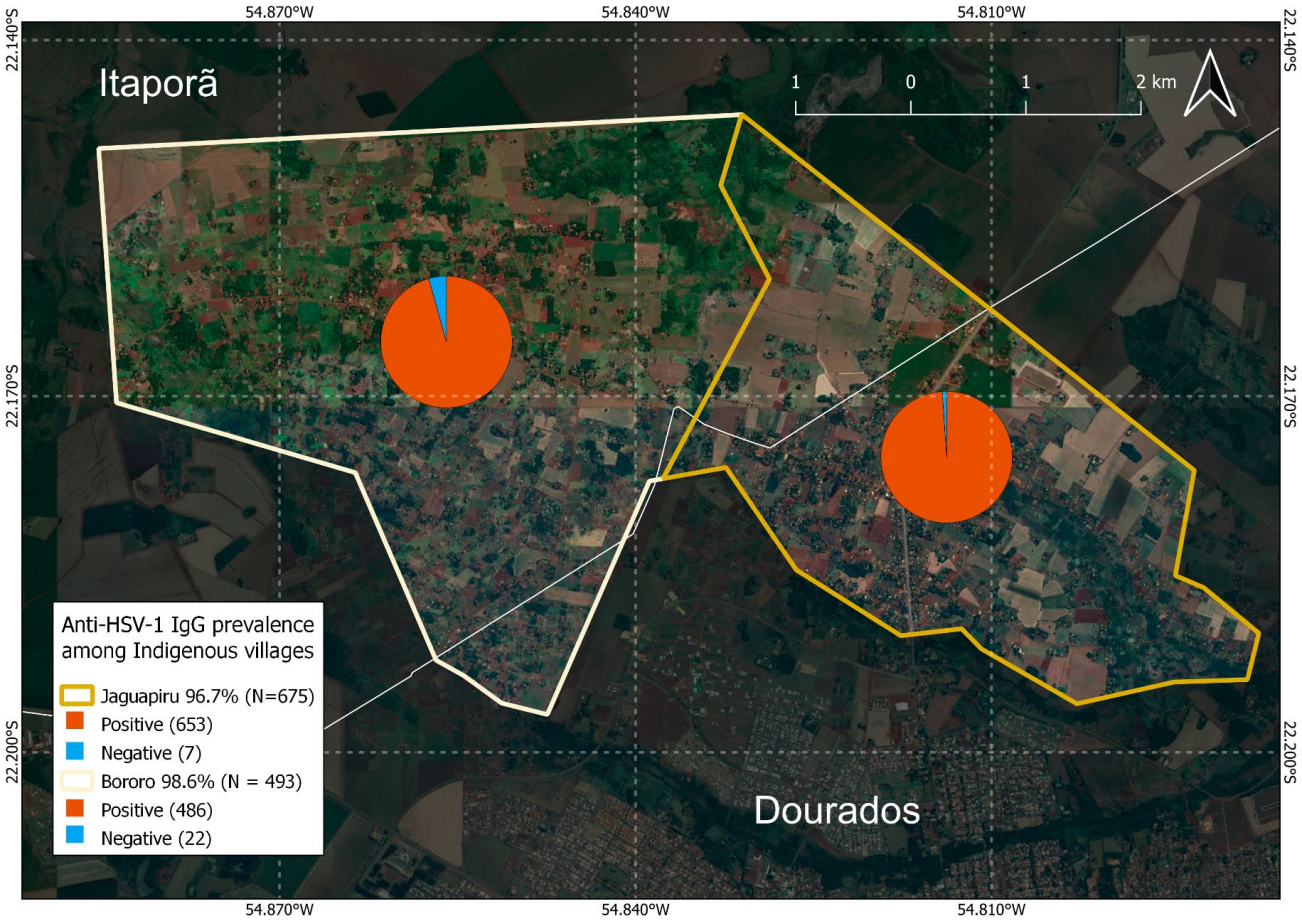
Distribution of anti-HSV-1 IgG positive and negative prevalence in Bororó (right) and Jaguapirú (left) villages (Dourados, Mato Grosso do Sul (MS), Brazil)

Bororó villagers (98.6%, 486/493) had a slightly higher HSV-1 IgG prevalence than Jaguapirú villages (96.7%, 493/653) (POR = 0.42(0.16–0.96; *p* = 0.07), showing a lower tendency in the Jaguapiru in relation to the Bororo, as shown in Table 1; Fig. 1.

The prevalence of anti-HSV-1 IgG in Indigenous people who declared to be retired was 97%, lived in another village previously was 97.7%, and had a family allowance of 97.8%, with no statistical significance. The use of cell phones to access health information was reported in 76.5% of participants, in which the HSV-1 IgG prevalence was 97% (POR = 0.23 (0.03–0.79); *p* < 0.05). Internet use was reported in 21.7% (POR = 0.51 (0.24–1.17) with an HSV-1 IgG prevalence of 96.1%. In participants that did not use cell phones or internet to access health information, the prevalence was 78.3% and 23.5%, respectively. In addition to these technological means, more than half of the participants used television (52%), while 48% did not.

**Fig. 2:**
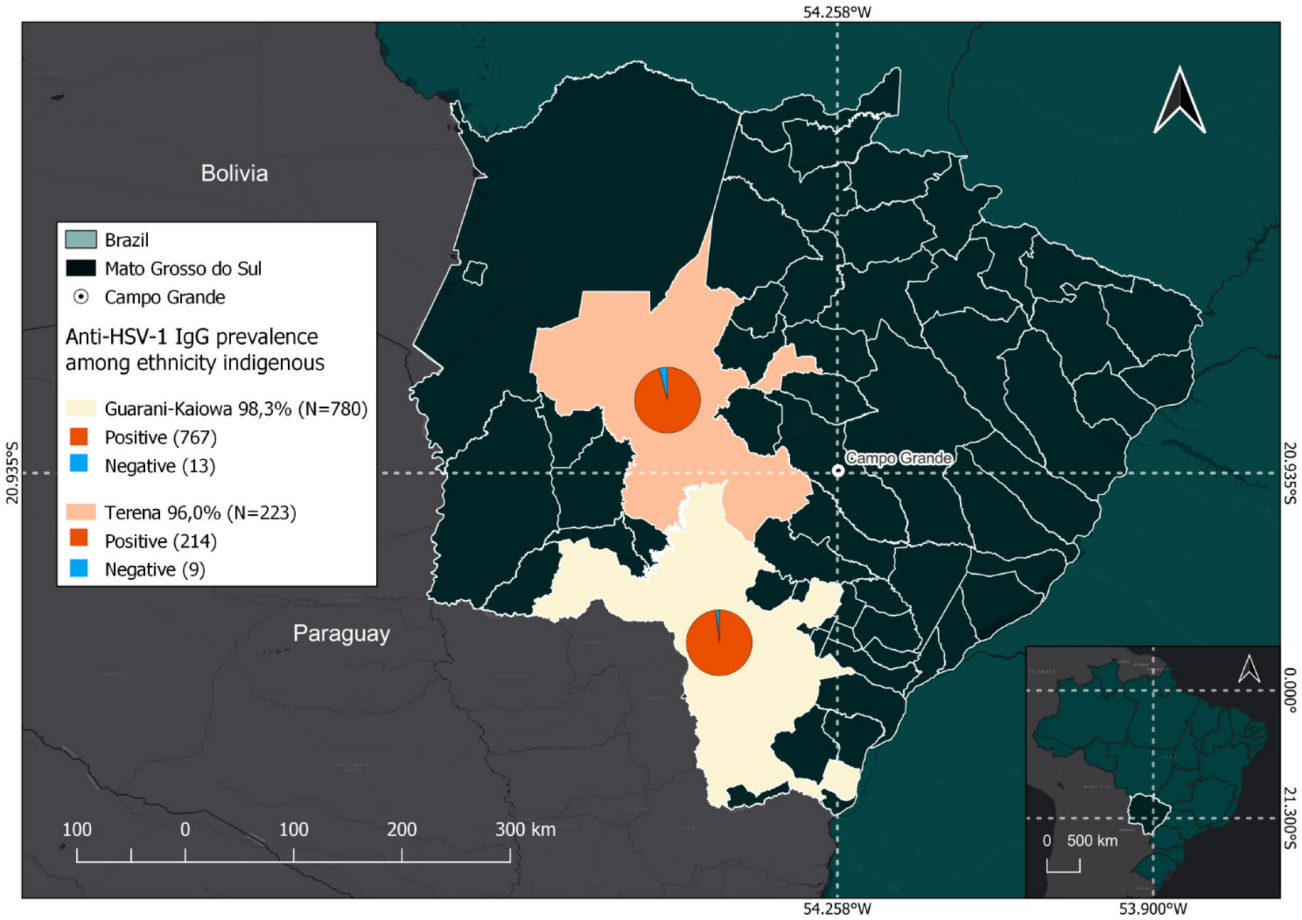
Positive and negative outcomes for anti-HSV-1 IgG serological tests among Indigenous Guarani-Kaiowá and Terena ethnicities and their geographical locations in Mato Grosso do Sul, Brazil

Regarding ethnicities, the anti-HSV-1 IgG prevalence in Guarani-Kaiowá was 98.3% (767/780) (POR = 2.53 (1.2–5.42); *p* < 0.05); Terena was 96% (214/223) (POR = 0.51 (0.23–1.20), and in other ethnicities (Guarani-Nhandeva, Kadiwéu and Guató) was 82.1%. Guarani-Kaiowá participants were 2.53 more likely to be positive for Anti-HSV-1- IgG than the other ethnicities with statistical significance (Fig. 2).

The prevalence of anti-HSV-1 IgG was found to be significantly influenced by Indigenous educational background. The prevalence in illiterate and elementary-educated participants was 98.2% (846/861) (POR = 1.14 (0.17–4.23), high-school-educated was 96.1% (246/256) (POR = 0.49(0.07–1.90) and college-educated was 92.2% (47/51) (POR = 0.23 (0.03–1.24) (Table 1), observing a decrease in the prevalence according to the educational level, and therefore a protective factor with higher educational levels.

Families earning > 5 minimum wages had a 100% prevalence of anti-HSV-1 IgG (10/10) accounting for 0.9% of the total Indigenous population; families earning < 1 minimum wages was 97.4% (666/684%), representing more than half of Indigenous population (58.8%); families earning 1–2 minimum wages was 98.6% (416/422) (POR = 1.87 (0.77–5.2); and families earning > 3 minimum wages was 92% (57/62) (POR = 0.25 (0.09–0.79). The p-value for family income was 0.004, which was statistically significant. Indigenous people who worked formally or informally tested 97.9% for anti-HSV-1 IgG.

Among those who reported condom use behaviors, the prevalence was 14.1% in those who reported ‘always’ used (POR = 0.35 (0.16–0.82), and 85.9% in those who reported ‘sometimes’ or ‘never’ used. In addition, the prevalences in those with risky behaviors and drug and alcohol history are reported in Table 1. The difference in prevalence for former prisoners, alcoholism, illicit drug use, syringe and needle sharers, smoking, and blood transfusions were not statistically significant. Sexual history, sexual activity, sexual intercourse with a partner who was an injection drug user, sexual intercourse with a partner who is an injecting drug user, sex workers, sexual intercourse with HIV carriers, STI history and single sexual partner. However, the prevalence of anti-HSV-1 IgG with respect to sexual preference was 100% in homosexual and 97.6% in heterosexual individuals. Although certain groups may have had higher prevalence rates, these differences were not statistically significant.

**Table 1:** Comparison of different variables associated with anti-HSV-1 IgG prevalence among Indigenous communities in Bororó and Jaguapirú villages (Dourados, Mato Grosso do Sul (MS), Brazil)

Age Median (Years)	30 [22, 40]*						
**Variable**	**N(%)**	**N Positive**	**N Negative**	**Prevalence (%)**	p-value**	POR*** (IC 95%)	
**Village**					0.07		
Jaguapiru	675 (57.8%)	653	7	96.7%		0.42(0.16–0.96)	
Bororó	493 (42.2%)	486	22	98.6%			
**Gender**					0.99		
Female	945(80.9%)	922	23	97.6%			
Male	223(19.1)	217	6	97.3%			
**Lived in another village previously**	869 (25.6%)	862	7	97.7%	0.99		
**Family allowance**	627(53.7%)	613	14	97.8%	0.68		
**Retired**	66 (5.7%)	64	2	97%	0.99		
**Technological devices**							
Cell phone	894(76.5%)	867	27	97%	0.005	0.23(0.03–0.79)	
Television	607(52.0%)	589	18	97%	0.36		
Internet	254(21.7%)	244	10	96.1%	0.14	0.51(0.24–1.17)	
**Ethnicities**							
Guarani-Kaiowá	780(66.8%)	767	13	98.3%	0.01	2.53(1.2–5.42)	
Terena	223(19.1%)	214	9	96.0%	0.15	0.51(0.23–1.20)	
Others	28 (22.1%)	23	5	82.1%			
**Education**					0.01		
Elementary School and Illiterate	861(65,9%)	846	15	98.2%		1.14(0.17–4.23)	
High School	256(21.9%)	246	10	96.1%		0.49(0.07–1.90)	
College education	51(4.4%)	47	4	92.2%		0.23(0.03–1.24)	
**Use of Condoms**					0.01		
always	165(14.1%)	156	9	94.5%		0.35(0.16–0.82)	
sometimes/never	1003(85.9%)	983	20	98%			
**Familiar income**					0.004		
< 1 minimum wage	684(58.8%)	666	18	97.4			
1 to 2 minimum wages	422(36.1%)	416	6	98.6%		1.87(0.77–5.2)	
> 3 minimum wages	62(5.4%)	57	5	92%		0.25(0.09–0.79)	
**Risk behaviors and drug/alcohol history**							
Former inmate	25(2.1%)	24	1	96.0%	0.99		
Alcoholism	275(23.5%)	269	6	97.8%	0.88		
Illicit drugs	43(3.7%)	42	1	97.7%	0.99		
Syringe and needle sharing	19(1.6%)	18	1	94.7%	0.96		
Smokers	146(12.5%)	143	3	97.9	0.94		
Transfusion of blood	97(8.3%)	95	2	97.9	0.99		
**Sexual history**							
Sexual intercourse with partner who is an injecting drug user	87(7.5%)	84	3	96.6%	0.80		
Sex worker	11(0.9%)	10	1	90.9%	0.65		
STI history	34(2.9%)	32	2	94.1%	0.46		
Sexual intercourse with HIV carrier	5(0.4%)	5	0	100%	0.99		
Single sexual partner	875(74.9%)	853	22	97.5	0.99		
Sexual intercourse with a partner who is a non-injecting illicit drug user	10(0.9%)	10	0	100%	0.99		
**Sexual orientation**							
homossexual	20(8.3%)	20	0	100%	0.99		
heterossexual	1150(98.5%)	1122	28	97.6%	0.93		

* Interquartile range; ** p-values from the chi-squared test; *** Crude Prevalence Odds Ratio; N: Number

In Table 2, the Guarani-Kaiowá ethnic group showed the highest rates of illiteracy (60.8%) and elementary school education (73.3%), which were different from the Guarani-Nhandeva and Terena ethnic groups, where the highest rates of college education were 35.3% and 3.9%, respectively. However, the Guarani-Kaiowá ethnic group had the highest rate with < 1 minimum wage (73.8%), and the Guarani-Nhandeva and Terena ethnic groups had the highest rates of income of 3–4 minimum wages (36.5%) and five minimum wages (10%), respectively. The Guarani-Kaiowá ethnic group had the lowest income and educational level.

**Fig. 3:**
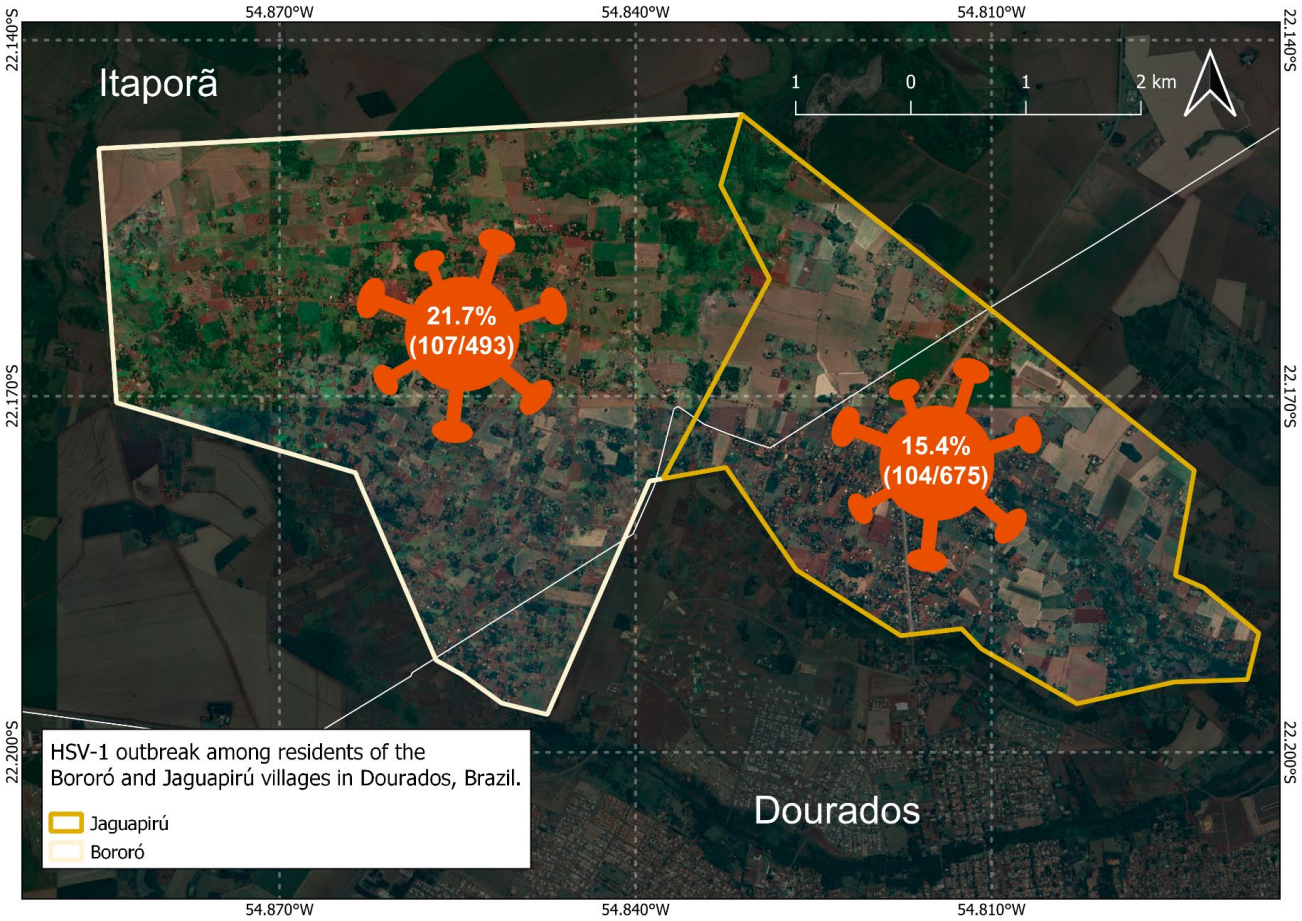
HSV-1 among residents of the Bororó and Jaguapirú villages in Dourados, Mato Grosso do Sul, Brazil

Among clinically symptomatic Indigenous individuals, anti-HSV-1 IgM was detected in 17.1%, 21.2%, 12.5%, and 22% of those exhibiting urinary problems, genital wounds, genital warts, and urethral discharge, respectively (Table 3; Fig. 3). Anti-HSV-1 IgM total was 20.8% and found in 21.7% (107/493) and 15.4% (104/675) of Indigenous individuals from Bororó and Jaguapirú villages, respectively. In total, 19.5% (150/767), 18.3% (42/223), and 15% (3/20) of individuals from Guarani-Kaiowá, Terena, and Guarani-Nhandeva, respectively, presented clinical symptoms and reacted to anti-HSV-1 IgM, which resulted in a very high prevalence of HSV-1 in these indigenous villages (Fig. 3). Positive serum anti-HSV-1 IgM tests underwent viral load detection and quantification for qPCR UL39. HSV-1 DNA with high viral loads were detected in participants aged 18–77, with an average of 31.3 (Table 4).

**Table 2:** Relationship between socioeconomic and education variables by ethnic group in Indigenous populations of Dourados, Mato Grosso do Sul (MS), Brazil

Variable	*N*	Guarani-Kaiowá		Guarani-Nhandeva		Terena	
Income		Prevalence	*p*-value*	Prevalence	*p*-value*	Prevalence	*p*-value*
			< 0.0001		< 0.0001		0.06
< 1 minimum wage	684	73.8%		2.9%		1.2%	
1 to 2 minimum wages	422	58.5%		26.8%		2.1%	
3 to 4 minimum wages	52	44.5%		36.5%		3.8%	
> 5 minimum wages	10	50.0%		30.0%		10.0%	
**Education**			< 0.0001		< 0.0001		0.08
Illiterate	102	60.8%		14.7%		2.0%	
Elementary school	759	73.3%		15.9%		1.1%	
High school	256	53.9%		27.0%		3.1%	
College education	51	47.1%		35.3%		3.9%	

* p-value estimated using Pearson’s chi-squared test

**Table 3:** Seroprevalences of anti-HSV-1 IgM in individuals exhibiting clinical symptoms in the Indigenous community, Mato Grosso do Sul, Brazil

Clinical symptoms	*N* (%)	*N* Positive	*N* Negative	Prevalence	*p*-value*
Urinary problems	123(11.7%)	21	102	17.1%	0.44
Genital Wounds	33(3.1%)	7	26	21.2%	0.99
Genital Warts	16(1.5%)	2	14	12.5%	0.65
Urethral discharge	100(9.5%)	22	78	22.0%	0.70

*p-values from the chi-squared test

**Table 4:** Viral load in participants from Indigenous populations with positive anti-HSV-1 IgM.

Age	Gender	Viral load (copies/mL)	CT	Clinical symptoms
27	F	5.99E + 02	40	NS
23	F	2.42E + 03	38	NS
22	F	4.23E + 03	37.2	NS
27	F	4.54E + 03	37.1	NS
33	F	5.22E + 03	36.9	NS
24	F	8.62E + 03	36.18	NS
39	M	1.07E + 03	35.87	NS
40	F	1.21E + 04	35.7	NS
26	F	1.29E + 04	35.6	NS
77	F	1.96E + 04	35	NS
23	F	5.22E + 04	33.6	NS
46	F	9.12E + 04	32.8	S
64	F	1,05E + 05	32.6	NS
35	F	1.09E + 05	32.54	NS
21	F	1.33E + 05	32.26	NS
26	F	1.39E + 05	32.2	NS
38	F	1.59E + 05	32	NS
34	F	1.59E + 05	32	NS
36	F	2.42E + 05	31,4	NS
33	F	3.44E + 05	30.9	NS
20	F	4.54E + 05	30.5	NS
54	F	6.91E + 05	29.9	NS
23	F	9.13E + 05	29.5	NS
35	M	3.95E + 06	27.4	NS
18	M	5.23E + 06	27	NS
26	F	6.01E + 06	26.8	NS
23	F	5.61E + 07	23.6	NS
23	F	6.45E + 07	23.4	NS
20	F	1.71E + 08	22	NS
39	F	2.27E + 09	18,3	NS
35	M	3.70E + 11	11	NS
37	F	2.12E + 12	8.5	NS
40	F	2.44E + 12	8.3	NS
20	F	2.61E + 12	8.2	NS
26	F	2.80E + 12	8.1	NS
18	F	3.92E + 12	7.62	S
27	M	4.41E + 12	7.45	NS
24	F	5.44E + 12	7.15	NS
20	F	5.75E + 12	7.07	NS
38	F	5.92E + 12	7.03	NS
38	F	6.48E + 12	6.9	NS
55	F	6.76E + 12	6.84	NS
19	M	6.95E + 12	6.8	NS
21	F	8.93E + 12	6.44	NS
25	M	9.85E + 12	6.3	NS
26	F	1.36E + 13	5.84	NS
30	F	1.42E + 13	5.78	NS
55	F	1.44E + 13	5.76	NS
26	F	2.12E + 13	5.2	NS
21	F	2.17E + 13	5.17	NS
27	F	3.27E + 13	4.58	NS
25	F	3.36E + 13	4.54	S

F: female; M: male; NS: no symptoms; S: symptoms

## Discussion

The prevalence of HSV-1 among Indigenous populations can be influenced by various factors that increase vulnerability and risk behaviors. These factors include cultural and social practices pertaining to age, sex, income, education, ethnic background, sex history, sexual partners, and condom use. Moreover, the sharing of contaminated sharp objects, alcoholism, and a history of STIs further contribute to the high prevalence of HSV-1 in this population. It has also been noted that the proximity to national and international borders as well as geographical location could increase the vulnerability of Indigenous populations to HSV-1 infections. Thus, it is imperative to consider these factors when developing effective strategies for STI prevention and control in these communities [38].

The overall prevalence of anti-HSV-1 IgM in the study was 20% (211/1053), varying from 21.7% (107/493) in Bororó to 15.4% (104/675) in Jaguapirú villages. The Guarani-Kaiowá, Terena, and Guarani-Nhandeva groups had anti-HSV-1 IgM prevalence of 19.5% (150/767), 18.3% (42/223), and 15% (3/20), respectively. The most frequent signs and symptoms of STIs were genital wounds, urethral discharge (22%), urinary problems (17.1%), genital sores (21.2%), and genital warts (12.5%). Among the 51 individuals with a history of STIs, 43 (84.3%) tested positive for anti-HSV-1. Further analysis revealed that three individuals with a previous clinical history of genital herpes were anti-HSV-1 positive. According to Sukik, in Latin America and the Caribbean, the virus isolation proportion was approximately 0.9% in genital urethral disease, and 10.9% in genital herpes [33]. These findings provide valuable insights into the prevalence of HSVs among individuals with a history of STIs [34].

Circulation of the virus was confirmed by qPCR: HSV-1 DNA was detected in 25.6% (54/211) of anti-HSV1 IgM-positive samples with viral loads ranging from 5,99E + 02 to 3,36E + 13, indicating a possible HSV-1 outbreak in these Indigenous communities. The high prevalence of HSV-1 is associated with morbidity, physical discomfort, cosmetic disfigurement and psychological distress. In 2014, Arizona had herpes outbreaks that led to exclusion from sporting events [39, 40]. Skin infections are a widespread problem among athletes, causing 8.5% of related adverse events. Wrestlers are particularly susceptible because of the constant skin-to-skin contact required during training and competition. A significant proportion (30%) of viral infections transmitted among high-school wrestlers are caused by HSV-1. These statistics highlight the importance of preventive measures to protect athletes’ health during potential outbreaks and high prevalence [41]. According to Amudha et al., nearly 20% of herpes infections manifest as vesicular or ulcerative lesions in the genital area. Moreover, more than 80% of these patients have no visible symptoms, which makes it easier for the virus to spread and infect healthy individuals. It is important to acknowledge and address these issues to ensure the implementation of effective prevention and management strategies [42, 43]. Based on the results that indicate a high prevalence of HSV-1 IgM in this population, it is reasonable to suggest that an outbreak of HSV-1 has occurred. However, there is no previous data on the prevalence of HSV-1 or other STIs in this population, to make a concrete claim of an HSV-1 outbreak. Furthermore, these findings suggest a significant rate of HSV-1 IgM reactivation or primary infection in this region, which may have contributed to the unexpected increase in infection. These results underscore the importance of continued HSV-1 monitoring in Indigenous communities to prevent its continued spread [44]. According to our analysis, a high percentage (97.5%) of Indigenous Brazilian people residing in Dourados, MS, had HSV-1 IgG antibodies. Furthermore, our research indicates that the prevalence of HSV-1 IgG antibodies is notably similar between the sexes, with females displaying a higher percentage (97.6%) compared to males (97.3%). It was observed that the majority of the people included in the study were women 80.9% (945/1168), while men constituted 19.1% (223/1168). This is due to the demographic location of the villages being located in difficult access to work, health care and, together with this, the social phenomenon on the days when the questionnaires were administered and the blood samples were taken, men, who are the financial providers of the household, were out of the villages due to work or hunting.

Our data also revealed that HSV-1 positivity was most common among those aged 22–40 years in the MS region, with an average age of 30 years at infection. According to Clemens, the prevalence rates for anti-HSV-1 were at 70.1% among 1,037 individuals in the general population of Brazil in 2010, with varying HSV-1 prevalence across different geographic regions of the country, with rates of 73.3% in Manaus, 68.6% in Rio de Janeiro, 62.1% in Porto Alegre, and 47.0% in Fortaleza. These findings provide important insights into the distribution of the virus within the population and may have significant implications for future public health interventions and prevention strategies [34].

A global HSV-1 infection prevalence is well-documented [45]. HSV-1 is responsible for a range of morbidities [39, 46], with orolabial herpes lesions as a typical clinical manifestation [39, 47]. Typically, lifelong, mostly asymptomatic infections are acquired during childhood via oral transmission [46]. However, mounting evidence suggests an HSV-1 epidemiological transition in Europe, North America [13, 47–49] and Asia [50], with an increasing number of individuals acquiring the infection through oral sex during adulthood [13, 47, 48]. The report demonstrated that HSV-1 infection is prevalent in Latin America and the Caribbean, with a seroprevalence of 67% in the global population [45]. Nearly 60% of children and 90% of adults are infected, with a high seroprevalence of 76.5% in adults [50, 51] and 96.2% in adults in Africa [52]. The seroprevalence of HSV-1 decreased from 62.0% in 1988–1994 to 57.7% in the year 1999–2004 in United States. Upon analyzing individuals infected with HSV-1 but not HSV-2, a higher percentage during 1999–2004, as compared to 1988–1994, were diagnosed with genital herpes (1.8% vs. 0.4%, respectively; *P* < 0.01), while the overall trend was positive, suggesting that there may be an upward trend in genital herpes among those solely infected with HSV-1 [51].

An epidemiological transition, defined as significant changes in the occurrence of the infection and/or its mode of transmission, has been observed in multiple Western countries, with HSV-1 becoming the primary cause of first-episode genital herpes, surpassing HSV-2 [13, 45, 47, 49, 53–55]. These data suggest the need for region-specific prevention and control strategies to effectively prevent the spread of the disease. According to a study on an Indigenous population in Australia, the HSV-1 seroprevalence was significantly higher, reaching 97.8%. Furthermore, all patients aged 16–24 years included in the study had positive antibodies against HSV-1, which is similar to the rates observed in developing countries where early adult infections approach 100% [56, 57]. In contrast, many developed countries, such as Japan [58], England [59], and the USA [60] report overall seroprevalence rates of HSV-1 to be less than 70%. This is supported by cross-sectional seroprevalence studies that indicate a significant decline in HSV-1 infections in developed countries over the past three decades [61]. This decrease is attributed to advancements in socioeconomic conditions, particularly sanitation and overcrowding, which have greatly reduced childhood acquisition of HSV-1. In a study in the Indigenous Australian population, the findings on HSV-1 were significant for public health decision-making for this population [62, 63].

A study conducted in villages in Dourados City (Mato Grosso do Sul) found that Bororó exhibited a moderately higher prevalence of HSV-1 than Jaguapiró at 98.6% and 96.7%, respectively (POR = 0.42 (0.16–0.96)). Bororó and Jaguapiru villages are predominantly inhabited by the Guarani-Kaiowá and Terena ethnicities [3, 4]. The Bororó population demonstrated higher adherence to the study, perhaps owing to limited access to healthcare services. Bororó village is geographically large, extending from Bolivia to the Miranda River in Brazil [64]. The population’s morbidity rate highlights precarious living conditions and largely comprises infections associated with basic sanitation, hygiene, and alcoholism. Both the Jaguapiró and Bororó Indigenous populations are a mix of families from multiple Indigenous communities, including Guanari-Kaiowá, Guarani-Nhandeva, Terena, Guató, Kadiwéu, Paraguayans, and regional Brazilians assimilated through interethnic marriages [64]. The results of our study indicated that the prevalence of anti-HSV-1 IgG among the Guarani-Kaiowá and Terena ethnicities was 98.3% and 96%, respectively, both of which were significant, with odds ratios of 2.53 (1.2–5.42) and 0.51 (0.23–1.2), respectively. In contrast, the prevalence of anti-HSV-1 IgG in other ethnicities was 82.1%, which was not statistically significant. Our findings suggest that individuals belonging to the Guarani-Kaiowá ethnicity are at a significantly higher risk of acquiring HSV-1 than those belonging to other ethnic groups in the study and are more numerous in the Bororo (72.3%; 405/560) village than in the Jaguapirú village (27.6%; 155/560). However, HSV-1 was more prevalent in the Terena ethnic group in the Jaguapirú village (88%; 92/104) than in the Bororo village (13%; 12/104).

Our study sheds light on the prevalence of HSV-1 among various ethnic groups in Brazil, providing valuable insights into the regional distribution of the disease. As shown in Table 1,the Guarani-Kaiowá ethnic group had the highest percentage of individuals with primary education (73.3%) and higher education (47.1%). However, the Terena and Guarani-Nhandeva ethnic groups had the highest rates of indigenous people with higher education (35.3% and 3.9%, respectively). The Guarani-Kaiowá also had lower income rates than other ethnic groups. This study revealed that the Guarani-Nhandeva and Terena ethnic groups had the highest levels of income and education. In addition, the Terena and Guarani-Kaiowá ethnic groups are located approximately 5 km from the city of Dourados, which allows them access to the urban center and free contact with non-indigenous populations [65]. A negative correlation was observed between the salaries received by indigenous people and the prevalence of HSV-1. When individuals earned between 1 and 2 minimum wages, their chance of acquiring the virus increased, with a prevalence of 98.6% (POR = 1.87 (0.77–5.2). Poverty, low income, limited access to medical care and limited information contribute to the high prevalence of HSV-1 in vulnerable populations, such as indigenous communities [64, 65].

The Indigenous population of Cape York in Australia is confronting unwarranted economic and social discrepancies such as elevated unemployment rates and cramped living conditions, along with lower household incomes and a reduced likelihood of completing secondary education [66]. These variables may be linked to the increased prevalence of HSV-1 infection in this vulnerable community. Further research is necessary to unravel the specific underlying factors that contribute to HSV-1 acquisition in this group.

With the advent of technology, electronic devices act as gateways for information and healthcare services, and access to these media has been linked to a higher HSV-1 prevalence [67–70]. In individuals who used cell phones, television, and the internet within Indigenous villages, the prevalence was 97% (POR = 0.23 (0.03–0.79)), 97%, and 96.1% (POR = 0.51 (0.24–1.17)), respectively, compared to non-device-users. Among these villages, 76.5% (867/894), 52% (589/607), and 21.7% (244/254) reported using cell phones, television, and the internet, respectively. However, those without access to communication media had a higher chance of acquiring HSV-1. Therefore, limited access to information and diagnostic services may be responsible for the higher prevalence of HSV-1 infection in Indigenous communities. Notably, the remoteness of some villages can affect media access, underscoring the importance of promoting disease prevention and awareness through improved communication channels [70]. The prevalence of anti-HSV-1 IgG among Indigenous individuals moving between villages was 97%. However, compared to those who refrained from travelling and migration, the risk of having anti-HSV-1 IgG was only 3% greater which was not statistically significant.

The results indicated a significantly higher prevalence of HSV-1 among smokers (97.9%) than among nonsmokers (2.1%), providing evidence that smoking is a risk factor for contracting HIV, STIs, and Tuberculosis, separate from poverty and alcohol use [71–73]. Further analysis revealed that Indigenous individuals who reported always using condoms (94.5%) were less affected than those who did not use or used sometimes (98%). Additionally, condom use was protective in nature in preventing STI transmission and statistically associated. The low condom use reported among the population may contribute to increased STI rates; even though saliva is the primary means of HSV-1 transmission, sexual transmission can also occur [74–79]. This study also found an association between a low frequency of consistent condom use and vulnerability to the transmission of other STIs among Indigenous migrant agricultural workers with same-ethnic partners, concurrent partners, and partners using illegal drugs. These findings emphasize the importance of promoting condom use as an effective measure for preventing STIs, especially in vulnerable populations [78–80].

According to our study, the presence of risky behaviors and a history of drug and alcohol use were linked to the acquisition of HSV-1. Our study showed a high prevalence of HSV-1 among individuals who had a former inmate status, received blood transfusions, engaged in illicit drug use, were alcoholics, and shared syringes and needle objects. We observed prevalence rates of 96%, 97.9%, 97.7%, 97.8%, and 94.7%, respectively; however, there were no statistically significant differences between them. HSV-1 infections associated with blood transfusions are more likely to occur in severely immunocompromised individuals, often leading to fatal generalized infections [27].

In this study, we identified additional risk factors contributing to a higher likelihood of acquiring and transmitting HSV-1. Sexual behaviors such as sex work, engaging in sexual encounters outside one’s cultural context, and experiencing repeated breakups are among these factors. Based on our analysis of Indigenous cultural behaviors, we found that certain sexual practices place individuals at a particularly high risk of HSV-1 infection [77, 81–83], such as sexual intercourse with a partner who is an injecting drug user, sex worker, STI history, HIV carriers and non-injecting illicit drug user, were risk factors for HSV-1 with rates of 96.6%, 90.9%, 94.1%, 100% and 100% of cases, respectively. These findings highlight the importance of targeted interventions and education to reduce the spread of HSV-1 in these populations. In sexual preference studies, a considerable proportion of Indigenous individuals who were identified as sexually active homosexuals exhibited a 100% prevalence rate for hsv-1, compared to their heterosexual counterparts who exhibited a prevalence rate of 97.6%. A study conducted in Melbourne, Australia revealed that 73.3% of HIV-seronegative homosexual males possessed HSV-1 antibodies [84], this study, indicating a significantly high prevalence (100%) for HSV-1 in the indigenous homosexual population in Brazil. These findings contribute to a better understanding of the impact of sexual preference on the prevalence of HSV-1 and can guide and prioritize future preventive measures. Despite our best efforts, the scope of this study was constrained due to the scarcity of blood samples required for molecular and phylogenetic analysis. These precious materials were procured from populations that are notoriously hard to reach, contributing to the challenges faced during our investigation.

## Conclusions

The prevalence of HSV-1 in Indigenous populations was significantly higher (97.5%) than in the general adult Brazilian population (70.1%). Our study also identified a high incidence of HSV-1 infection and circulation of HSV-1 DNA within the Jaguapirú and Bororú village populations. The discovery of HSV-1 IgM suggests that reactivation or primary infection of HSV-1 may have caused an outbreak of the virus in the Bororó and Jaguapirú Indigenous populations, *due to its high HSV1 prevalence*. It is important to emphasize that HSV-1 is a latent virus that can be reactivated and, in some cases, cause serious neurological complications. If left untreated, widespread and disseminated HSV-1 infections can lead to high mortality rates in high-risk groups. Several socioeconomic factors, sexual behaviors, and drug use contribute to the increased HSV-1 prevalence in Indigenous population in Brazil. As such, culturally appropriate intervention programs are necessary to eliminate health access barriers faced by Indigenous populations. Additionally, public health policies should aim to promote information on HSV-1 infections and therapeutically prevent and control the virus among the Indigenous populations in Brazil. Achieving these goals requires a respectful and culturally sensitive approach, including the promotion of appropriate condom usage. Our research offers valuable insights into the prevalence and potential risk factors contributing to HSV-1 infections among Indigenous populations in Brazil, providing more effective public health policies and interventions. In conclusion, it is important to acknowledge that the health requirements and susceptibilities of Indigenous Peoples are diverse, given that they represent unique and multifaceted communities inhabiting varying environmental and social conditions.

## Data Availability

The data presented in this study are available upon request from the corresponding author. All the data from this study are reported in this article.

## Abbreviations

HSV-1: Human Alphaherpesvirus 1
MS: Mato Grosso do Sul
IBGE: Geography and Statistics
FUNAI: National Indian Foundation
STIs: Sexually transmitted infections
HIV-1: Human immunodeficiency virus-1
UFGD-MS: University of Grande Dourados
SIHD/MS: Special Indigenous Health District of Mato Grosso do Sul
IgM: Anti-HSV-1 (gG1) type M
IgG: Anti-HSV-1 (gG1) type G
POR: Prevalence Odds Ratio
